# Intra-Thoracic New Growths

**Published:** 1908-02-22

**Authors:** Frederick Taylor

**Affiliations:** Consulting Physician to Guy's Hospital, Physician to the Seamen's Hospital, Greenwich.


### Hospital Clinics.

### INTRA-THORACIC NEW GROWTHS.

By FREDERICK TAYLOR, M.D., F.R.C.P., Consulting Physician to Guy's Hospital, Physician to the Seamen's Hospital, Greenwich.

(Abstract of a lecture delivered at the London School of Clinical Medicine, February 5, 1908.)

The subject of intra-thoracic growths is a large one, and I shall therefore only deal to-day with the clinical point of view, as that which appeals to you most. I shall not enter into questions of pathology, except as regards certain points on which information is necessary to understand the diagnosis and treatment.

Intra-thoracic growths, from a clinical point of view, may be said to include lymphadenoma, whether part of Hodgkin's disease or not; sarcoma; and carcinoma. Other conditions must be mentioned, such as hydatid and other cysts; but they are so rare that we need not consider them to-day. One cannot, however, exclude from consideration and discussion that other very common condition, aneurysm of the aorta or of the large trunks springing from it. As a source of pressure on the thoracic contents, it is much more often met with than are actual growths; but I shall not deal with it in detail except in so far as is necessary to distinguish its symptoms from those of the latter.

Neoplasms in the thorax may be primary or secondary. Of primary growths, lymphadenoma may develop in the bronchial glands as a part of a general (Hodgkin's) lymphatic disease. In such an event the enlarged spleen, in which there may be definite masses of growth, and the enlarged glands may be recognised clinically, and naturally the suspicion will arise that any symptoms in the chest may be due to lymphatic disease there too. Sarcoma, too, is very frequently primary in this situation; and carcinoma may be, as, for instance, when it arises in the epithelial lining of a bronchus. More frequently carcinoma is a secondary growth, and it then occurs in the bronchial glands or in the lung itself. The primary focus may be in the breast, abdomen, tongue, neck, or, less commonly perhaps, in the limbs.

The production of the signs and symptoms which give evidence of malignant growths in the thorax is at first almost entirely due to pressure effects on the neighbouring parts. Although we believe that in many cases of cancer there is what is called cancerous cachexia, which is evident in the aspect of the patient, in the majority of cases this is a late result; and we cannot exclude cancer as the cause of symptoms of intra-thoracic pressure because the patient does not show loss of flesh and debility.

Cancer may occur in various positions in the chest. First, we may consider the central position, in which it is necessarily related to very important structures, such as the aorta and great vessels, the nerves, pericardium, and even the thoracic duct. This group may be further divided into two others—one that of growths affecting mainly the bronchus and root of the lung, the other that of those which obstruct the great arteries or veins. Next to these we shall have to consider the case of malignant growths which occupy the lower part of the lung, on either side or on both. Then we come to a much less common situation for growth, the upper part of the lungs. Frequently, of course, there are modules of secondary growth disseminated through both lungs, back and front, high and low, without any one mass large enough to cause localising pressure symptoms. Finally, the pleura may be involved, and the surface of the lung.

The most familiar form in which we are acquainted with intra-thoracic new growth (large enough to cause pressure) is that in which there is obstruction to the flow of blood through the superior vena cava. As a result of this the blood cannot reach the right heart from the head, neck, and arms, and this causes swelling, and later on definite œdema of those parts. The collateral channels of circulation with the tributaries of the inferior vena cava are enlarged and developed in a manner characteristic of this lesion. The veins of the mammary region are dilated, and communicate with the superior epigastric veins, by which the blood reaches the inferior vena cava. A certain amount of tortuosity can then be seen, especially in the upper part of the chest. Often this is evident as a number of quite small tortuous venules all over the front of the chest, a condition distinct from that seen when it is the inferior cava which is obstructed, as after typhoid fever, puerperal sepsis, or pelvic inflammation. In these latter cases there is generally one very definite large tortuous vein, in which it can be shown very easily that the blood is passing upwards. This single vein is prominent and distinct, and I wish to emphasise the fact that it is a condition not so marked in obstruction of the superior vena cava.

It must be admitted that this evidence of venous obstruction is not always found; that depends, no doubt, upon the rapidity of the growth (or aneurysm), and upon its exact position. Growths, however, have as a rule a very pronounced effect on veins, because they not only compress them, but also grow into them. For that reason, obstruction of the venous return is more often seen in cases of new growth than in cases of aneurysm. Arteries, which are commonly the seat of aneurysm, resist growths much better, and it is not very rare to see a patent and contractile artery tunnelling and unobstructed by a malignant growth. To this rule, however, there are exceptions.

Let me now take the root of the lung. Here both aneurysm and new growth occur. The latter may begin in a bronchus as an endothelial carcinoma of the mucous membrane, or it may be a carcinomatous or a lymphadenomatous gland outside the bronchus, and pressing on it. In either case there is obstruction of the airway, with the symptoms that arise therefrom. Nor must it be forgotten that from a carcinoma there may be hæmorrhage, though hæmoptysis from this cause is not common. There are three very definite symptoms of compression of the root of the lung.

(1) There is diminution of the vesicular murmur in the affected lung. When diminished air entrance is marked, one finds not only that, but also an exaggerated vesicular murmur on the healthy side: this makes the contrast so much greater that the condition ought never to be overlooked. At the some time the lung is resonant in this early stage, a combination which is to be regarded as very significant when it is found over an extensive area of lung, and when acute inflammatory diseases can be excluded. (2) As time goes on the lung becomes collapsed, in consequence of the absorption of air from its interior. There used to be current what was called the ball-valve theory of this absorption, but I do not think it is seriously advocated now. There is no necessity for such an explanation of the facts, for stagnant air will be absorbed by the blood-vessels, as for instance from the subcutaneous tissue, and from the intestines. If, then, there is sufficient pressure on a bronchus to stop the air current, the whole lung may collapse by absorption of its contained air. In this stage there is dullness to percussion. (3) Now follows a very important change which may cause doubt or mistake unless you have it fully before your minds. This is the subsequent dilatation of the bronchi, with production of bronchiectasis. Probably the secretion of the occluded bronchi accumulates, becomes infected with microorganisms from the air, and leads to the formation of pus, and later to dilatation. The result is this, that in a long-standing case of obstruction of a bronchus you may get at one or other part of the lung a tympanitic note, possibly, with faint bronchial breathing, if one of these cavities lies under the chest wall at the place of examination, and still communicates with the air.

It must not be forgotten that some cases of malignant disease are associated with pleural effusion, the physical signs of which are the same as those of collapsed lung with growth. The distinction can often be drawn only by the use of the exploring needle, repeated if necessary; and it does not follow because pleural fluid is aspirated that a malignant tumour does not also exist.

Those, then, are some of the important developments that may follow mediastinal growths at the root of the lung. Now let us take up the case of one occupying the lung itself, perhaps a large part of it. The physical signs are then dullness, loss of vocal resonance, of tactile fremitus, and of expansion of the chest: in fact, they give the clinical picture of pleural effusion. I have often made the remark that the diagnosis of this latter condition is really made from the history of the patient rather than from his physical signs, because the signs which are accepted as those of pleural effusion are not conclusive of that condition; they are really only those of absence of the healthy lung structure, and are equally produced by growth or any other condition which causes this absence. The diagnosis of pleural effusion is made, apart from the history of an antecedent acute pleurisy, on the much greater frequency of that lesion over the other conditions which may give rise to the same signs. Malignant disease of the lung is not the only lesion which may thus be confounded with pleural effusion: cancer of the liver, or a hydatid cyst may push up the diaphragm and compress the base of the lung.

The resemblance of these cases to pleural effusion is so close that many of them are thus diagnosed. Sometimes the chest is opened surgically before it is discovered what the condition really is, and the mistake is a natural one, because of the much greater frequency of effusion. If the symptoms of effusion in any patient are of some standing, if they have not been led up to by a history of an acute onset, and if one or two exploratory punctures have been unsuccessful, it is right to bear malignant disease of the lung in mind.

Cancer, in the way it attacks certain patients, may sometimes simulate acute pleurisy very closely. A sudden rigor, acute pain in the side, cough and expectoration have been known to occur in a patient who had had a malignant tumour removed from his tongue, and death followed in a few weeks. Although in this case there was no post-mortem examination, it is reasonably sure that there were secondary masses in the chest. The diagnosis from tuberculosis, too, is not always simple: the signs of a cavity at the apex, with crepitations and pyrexia, and even hæmoptysis, may obscure the presence of a growth and be put down to that disease. Disseminated nodules may produce the signs of general bronchitis; there may be crackles and râles all over both lungs.

Cancer of the surface of the lung is almost certain to produce pleurisy. In connection with what I have told you about the distinction between these two conditions, the old dictum that the former causes no rise of temperature does not hold good. When a cancer is infected by organisms from the outside, rise of temperature would not be unexpected; but it is not necessary that any such accident should take place. Cancer may be associated with continuous high temperature without any such infection. A soldier came under my care last year in Guy's Hospital who had tumours in his liver which were supposed, on inspection at an operation, to be gummata; he had also continuous pyrexia. Twelve months later his temperature was as high as ever, in spite of anti-syphilitic treatment, and it was eventually demonstrated by operation and by post-mortem inspection that the growths were malignant.

Perhaps the teaching to which I refer is not now so prevalent as it used to be; certainly malignant disease of itself may be the cause of high temperature. Lymphadenoma, too, may be associated with pyrexia. It often happens in this disease that there are periods of two or three weeks of high temperature, followed sometimes by intervals of low temperature.

The question of treatment I shall not enter into. The possibility of removal of mediastinal cancer is scarcely yet entertained, and there is little to be hoped for in the application of the Röntgen rays to tumours as deep-seated as these.

